# Supplementary material for: Reduction in hospitalised COPD exacerbations during COVID-19: A systematic review and meta-analysis
Source: PLoS One. 2021 Aug 3;16(8):e0255659. doi: 10.1371/journal.pone.0255659 (PMC8330941; doi:10.1371/journal.pone.0255659)
Supplement: S1 Appendix — (DOCX) [file pone.0255659.s002.docx]

**S1 Appendix:**

**S1 Table: Medline search strategy.**

Database: Ovid MEDLINE(R) and Epub Ahead of Print, In-Process, In-Data-Review & Other Non-Indexed Citations and Daily

Search Strategy:

--------------------------------------------------------------------------------

1 Coronavirus Infections/

2 Coronavirus/

3 COVID-19.mp. [mp=title, abstract, original title, name of substance word, subject heading word, floating sub-heading word, keyword heading word, organism supplementary concept word, protocol supplementary concept word, rare disease supplementary concept word, unique identifier, synonyms]

4 SARS-CoV-2/

5 1 or 2 or 3 or 4

6 Pulmonary Disease, Chronic Obstructive/

7 (obstructive adj3 (lung or pulmonary or respiratory or bronchopulmonary)).mp. [mp=title, abstract, original title, name of substance word, subject heading word, floating sub-heading word, keyword heading word, organism supplementary concept word, protocol supplementary concept word, rare disease supplementary concept word, unique identifier, synonyms]

8 (COAD or COBD or COAD or COBD or COPD).mp. [mp=title, abstract, original title, name of substance word, subject heading word, floating sub-heading word, keyword heading word, organism supplementary concept word, protocol supplementary concept word, rare disease supplementary concept word, unique identifier, synonyms]

9 Emphysema/

10 Patient Admission/

11 Hospitalization/

12 exacerbation*.mp. [mp=title, abstract, original title, name of substance word, subject heading word, floating sub-heading word, keyword heading word, organism supplementary concept word, protocol supplementary concept word, rare disease supplementary concept word, unique identifier, synonyms]

13 exacerbate*.mp. [mp=title, abstract, original title, name of substance word, subject heading word, floating sub-heading word, keyword heading word, organism supplementary concept word, protocol supplementary concept word, rare disease supplementary concept word, unique identifier, synonyms]

14 6 or 7 or 8 or 9

15 10 or 11 or 12 or 13

16 14 and 15

17 5 and 16

**S2 Table. Quality assessment:**

**Details of the quality of Cohort studies**

| First author | Population representative | Sample size  adequate | Confounders | Statistical  analysis | Missing  data | Methodology  of the  outcome | Objective  assessment | OVERALL  (0-3, higher score =  lower risk of bias) |
| --- | --- | --- | --- | --- | --- | --- | --- | --- |
| Baeza-Martíneza et al. 2020 | 1 | 0 | 0 | 0 | 2 | 0 | 0 | 0.4 |
| Berghaus et al. 2020 | 2 | 1 | 1 | 2 | 1 | 3 | 2 | 1.7 |
| Chan et al. 2020 | 3 | 1 | 1 | 3 | 1 | 3 | 2 | 2 |
| Faria et al. 2021 | 3 | 1 | 2 | 3 | 2 | 3 | 3 | 2.4 |
| González et al. 2021 | 3 | 2 | 1 | 3 | 1 | 2 | 3 | 2.1 |
| Helgeland et al. 2021 | 2 | 0 | 1 | 3 | 1 | 3 | 3 | 1.8 |
| Hu et al. 2020 | 2 | 2 | 1 | 3 | 1 | 3 | 1 | 1.8 |
| Huh et al. 2021 | 2 | 1 | 1 | 2 | 1 | 2 | 2 | 1.5 |
| Kyriakopoulos et al. 2021 | 2 | 1 | 2 | 3 | 2 | 2 | 3 | 2.1 |
| McAuley et al. 2020 | 3 | 2 | 1 | 3 | 3 | 3 | 3 | 2.5 |
| Stohr et al. 2020 | 2 | 3 | 1 | 3 | 1 | 2 | 3 | 2.1 |
| Sykes et al. 2020 | 2 | 1 | 0 | 3 | 1 | 3 | 2 | 1.7 |
| Tan et al. 2020 | 2 | 1 | 1 | 2 | 1 | 2 | 3 | 1.7 |

0 = definitely no (high risk of bias); 1 = mostly no; 2 = Mostly yes; 3 = definitely yes (low risk of bias)
